# Integrated Care in England – what can we Learn from a Decade of National Pilot Programmes?

**DOI:** 10.5334/ijic.5631

**Published:** 2021-10-29

**Authors:** Richard Q. Lewis, Kath Checkland, Mary Alison Durand, Tom Ling, Nicholas Mays, Martin Roland, Judith A. Smith

**Affiliations:** 1 Nuffield Trust, UK; 2 Centre for Primary Care and HSR, University of Manchester, UK; 3 Policy Innovation and Evaluation Research Unit, London School of Hygiene and Tropical Medicine, UK; 4 RAND Europe, UK; 5 University of Cambridge, UK; 6 Health Services Management Centre, University of Birmingham, UK

**Keywords:** integrated care, pilots, evaluation, pioneers, vanguards

## Abstract

**Introduction:**

For more than a decade the English NHS has pursued integrated care through three national pilot programmes. The independent evaluators of these programmes here identify several common themes that inform the development of integrated care.

**Description:**

The three pilot programmes shared the aim of better coordination between hospital and community-based health services and between health and social care. Each programme recruited local pilot sites that designed specific interventions to support inter-professional and inter-organisational collaboration.

The pilots were highly heterogenous and results varied both within and between the three programmes. While staff were generally positive about their achievements, pilots had mixed success especially in reducing unplanned hospital admissions. Common facilitators to achieving pilots’ objectives included effective senior leadership and shared values, simple interventions and additional funding. Barriers included short timescales, poor professional engagement, information and data sharing problems, and conflicts with changing national policy.

**Discussion:**

There was little stable or shared understanding of what ‘integrated care’ meant resulting in different practices and priorities. An increasing focus on reducing unplanned hospital use among national sponsors created a mismatch in expectations between local and national actors.

**Conclusion:**

Pilots in all three national programmes made some headway against their objectives but were limited in their impact on unplanned hospital admissions.

## Introduction

For more than a decade, the English national health service (NHS) has implemented a series of national pilot programmes designed to deliver better integrated care. This reflects the concern of successive governments to address the poor coordination of services which is perceived to be a significant problem within the NHS and between health and social care services. The objectives of integrated care include improving the clinical and cost-effectiveness of care by removing duplication, avoiding care ‘gaps’ and improving patient/user and informal carer experience.

Each of the national pilot programmes has followed hard on the heels of (or even overlapped with), its predecessor and each has had similar aims, design and implementation. Taken together, they represent a determined effort to change the way in which health and care services are delivered, albeit specific objectives in terms of service change are not always detailed or clear. Furthermore, given that each initiative has been independently evaluated, they collectively offer an opportunity to draw lessons for the future development and evaluation of integrated care, at a time when the government has firmly committed itself to a policy of system and service integration in England after a long period emphasising provider competition [1,2,3,4,5,6,7,8,9,10,11,12,13].

In this article, key members of the national evaluation teams of the three most prominent integration pilot programmes have collaborated to compare and synthesise findings from their studies. This was done through iterative discussions among the authors. In addition, the authors have variously participated in a number of seminars intended to share learning from the pilot programme evaluations and the debate generated at these events has also informed the findings set out in this article. Lastly, we have also drawn on a small number of other published evaluations of individual pilot sites (or groups of sites) within the wider programmes whose findings highlight important aspects of service integration in the English context. The paper thus represents a synthesis of empirical findings rather than a systematic review of the wider literature.

## Piloting Integrated Care in England

Three major national pilot programmes for integrated care have been initiated within the NHS since 2008 (Integrated Care Pilots, Integrated Care and Support Pioneers and New Care Model ‘Vanguards’). Broad descriptions of these pilot programmes are provided in ***Table 1***.

**Table 1:** Description of the three integration pilot programmes.


INTEGRATED CARE PILOTS (ICPS)	INTEGRATED CARE AND SUPPORT PIONEERS (PIONEERS)	NEW CARE MODEL VANGUARDS (VANGUARDS)

Programme launched in 2008 following the NHS Next Stage Review. Sixteen pilots appointed in 2009 designed to support care integration. Loose collection of aims including care closer to the user, greater continuity of care and a reduced use of hospital care. As NHS finances became constrained nationally, the focus shifted to aim of reduced cost Deliberately heterogenous mix of pilots in terms of: - scale (from a single GPpractice with a population of 6300 to a broad rangeof services for a population of 500,000). - Target groups (somepilots focused on single cohorts such as elderly people, otherson diseases such as dementia and diabetes). A sub-setof 6 pilots focused on ‘case management’ interventions for olderpeople at risk of admission. - Integration focus (mainly horizontal integrationwithin community- based services with one pilot vertically integrating GPand hospital care) National programme support including project management resources and modest central funding for pilots.	Two waves of pilots launched since 2013 (14 pilots and 11 pilots respectively) Relatively homogenous goals including the ‘triple aim’ and person-centred care. Focus on three overlapping cohorts: older people with multiple long-term conditions; high service users, those at risk of hospital admission Pilots pursued a broadly similar range of interventions. Over time these narrowed to focus on: care navigators, multi-disciplinary teams, care planning and a single point of access for service users Pilots designed around horizontal and vertical integration of NHS and social care providers with a small number of pilots explicitly led by Local Authorities. Limited national programme support with modest central funding for pilots	Launched in 2015 with the aim of using pilots to define new ‘models’ of care which could subsequently be spread more widely. Focus on horizontal and vertical integration between sectors. Nationally prescribed range of three different integration ‘types’: - 9 Primary and Acute Care Systems(joining GP, hospital, community health and social care providers) - 14Multispecialty Community Providers (moving hospital specialists into community settings) - 6Enhanced Care Homes (integration of care homes and wider careservices). Local discretion over how these models were to be designed and implemented with expectation that new models to be scaled across the NHS. Multiple new services implemented with no clear differentiation between the three ‘types’ Over time, increased national focus on reducing use of hospital services Extensive national support programme and significant additional funding


All three pilot programmes shared similar high-level aims, such as breaking down perceived barriers between service providers, improving the ‘user-centredness’ of care and providing more services in a community setting. However, there were differences between the programmes and between the pilot sites within those programmes in the way that ‘integrated care’ was interpreted and how pilots were structured. This led to a high degree of heterogeneity among the pilot sites in terms of their scale of operation, the priorities emphasised, patient groups targeted, interventions implemented and types of organisations involved.

In part, this heterogeneity was a deliberate policy choice, with a degree of ‘bottom-up’ design of precise objectives and delivery mechanisms being built into the programmes and an expectation that the rest of the country would learn from their experiences. It might be said that national agencies decentralised the responsibility for addressing the longstanding problem of service coordination (a problem that the centre had persistently failed to resolve itself). Central agencies, for their part, made commitments to resolve those barriers identified where this required national action [14]. However, in practice there was little compelling evidence that significant new regulatory changes to facilitate integration were introduced in response to the pilots’ experiences.

All three programmes selected pilot sites from a larger number of volunteer areas. The selection criteria were generally permissive (focusing as much on the coherence of applications and evidence of past joint working as on the content of the proposal). However, it is notable that the degree to which pilots were ‘shaped’ by their NHS sponsors tended to increase successively. For example, the Integrated Care Pilots (ICPs) were perhaps the most diverse with the Vanguard pilots defined from the outset into three broad ‘types’ of integration model (in addition to the three Vanguard types highlighted here, the latter stage of the programme also included two additional models largely focused on the reorganisation of hospital-based services which have been excluded from this analysis as not directly related to service integration).

Notwithstanding the variation between the individual pilots, a number of common features could be identified within and across the programmes. First, there was a degree of congruence in terms of the patient/user populations targeted – many pilots focused their efforts on services for frail older people and those with multiple long-term conditions (although with needs largely defined by the service providers). There was also clustering around the types of interventions developed for these patient/user groups, which often included multi-disciplinary team meetings for care planning, the use of a ‘case manager’ to coordinate care around individuals and the application of risk stratification techniques to identify patients most at risk of unplanned hospital admission. At the same time, there was no obvious evidence that each programme built on the experiences of its predecessor in terms of refining models of care. This may reflect the modern tendency within government to deliver policy change through disconnected projects rather than as an ongoing process of policy evolution [15].

This project focus could also be seen in the existence of national sponsors and the central provision of programme management support to the local sites, a common feature shared by all three pilot programmes. However, this support varied in intensity. ICPs received some funding to support their activities together with the support of management consultants to assist with project planning and management. Pioneers received relatively modest financial support but had a designated ‘account manager’ provided by NHS England whose role was to support pilot participants (and provide information back to NHS England). In addition, Pioneers had access to an online platform containing information deemed to be helpful. In contrast, Vanguards received comparatively lavish amounts of additional funding, including funds to commission local evaluations (total funding across the programme was estimated by the National Audit Office to be £329M over three years [16]) and the support of a national support programme (which among other things supported local programme design). All initiatives were given access to networking events to share experiences.

While individual pilots developed a broad range of local success criteria, there was a general expectation among programme sponsors, usually shared by pilots themselves, that integrated care would result in a reduction in the level of unplanned hospital admissions. As the financial context for the NHS worsened following the onset of wider economic austerity, this expectation was heightened and the relative importance of other objectives such as improving patient experience or clinical quality diminished.

## Key themes identified through evaluation

The key themes that emerged from the independent evaluations, drawn largely from the authors’ published reports and refined through discussion, are summarised in ***Table 2***. The similarity in the facilitators and barriers identified as shaping each programme is striking. Common to all was the perceived importance of shared values within the initiative, effective leadership of the pilot, the availability of resources and strong, pre-existing local relationships between stakeholders.

**Table 2:** Comparison of significant pilot experiences and outcomes (extracted from publications of the national evaluation teams and selected studies) [1,2,3,4,5,6,7,8,9,10,11,12].


THEME	ICPS	PIONEERS	VANGUARDS

Facilitators	Effective senior leadership Shared values and vision Strong, pre-existing relationships locally Staff engagement Low complexity interventions (compared to high complexity) Specific education/training interventions Co-location of staff Availability of funding and other resources	Effective cross organisation and professional relationships Effective leadership Kudos as part of a pilot programme Shared vision and values Lack of organisational complexity (especially if shared boundaries) History of successful integrated care Availability of resources Staff engagement and ‘ownership’	Development of relationships with national programme team Multi-modal communications Strong local and national leadership Access to expert knowledge and skills Good level of funding Perception of a licence and platform to do things differently as a result of being part of a high-profile national programme

Barriers	Complexity of organisations and interventions IM&T issues and information governance concerns Poor communication Poor professional engagement (especially GPs) Erosion of professional identity ‘Red tape’ Wider NHS financial pressures Lack of resources and high existing workforce pressure Conflicts with new national policy context	Financial constraints and high existing workforce pressure IM&T issues and information governance concerns Limits to local freedom to innovate Limited national support to tackle systemic barriers Difficulty breaking down professional and organisational roles and culture Leadership tensions between organisations Engagement and commitment of GPs Conflicts with new national policy context	Continuation of standard national regulation and oversight Lack of high quality data and issues with information governance, inter-operability of systems and data sharing Short timescales and expectations of rapid progress (especially against government targets)

Impact on hospital activity	Significant increase in unplanned admissions and reductions in elective elective inpatient and outpatient care. More marked increase in unplanned admissions for case management sites. Overall costs of hospital care reduced by 9% (statistically significant) for case management patients.	A modest impact on unplanned admissions to hospital, with Wave 1 pilots experiencing a lower increase than non-Pioneers. However this was only statistically significant in Year 1 and not in Year 2. Significant variation found between pilots and within pilots.	Vanguards slowed the rise in unplanned admissions compared to controls. Over three years a significant 4.2% reduction in those admissions found for Enhanced Care Home pilots (increased over time and became statistically significant in third year and overall). MCP/PACS significant 3.1% reduction in Year 3 but not significant over whole period. No overall reduction in bed-days Sites had higher unplanned admissions and bed days than controls in two years prior to start of pilot. Impacts most visible in sites which had previously been Pioneers

Impact on patient experience	Mixed response. No more likely to have discussions about how to deal with health problems, more likely to have care plans In case management sites: more clarity regarding discharge; less likely to have been given wrong medicine. But also less likely to be able to see clinician of choice and fewer felt opinions and preferences taken into account.	Data are being collected on MDT caseload patients’ experiences of care received, and on the impact of being on an MDT caseload on health and quality of life.	No systematic study of patient experience across the programme. Individual Vanguards procured individual evaluations, but quality mixed.

Impact on staff experience	Staff reported improved team working and communication; increase of breadth and depth of their job; more responsibility; more interesting jobs; improvements to patient care.	Currently completing data collection on strategic level managers’ and operational as well as front line staff perceptions of health and social care integrated, community-based MDT working.	Staff reported increased job satisfaction associated with the feeling that they had licence to innovate and were part of a high-profile national programme.


These are hardly surprising given that they might be considered essential ingredients of any attempt to deliver a change to the delivery of health and care services. Sites in all the programmes also found it easier to make progress when implementing relatively discrete interventions rather than complex, multi-factorial system changes. Fewer participating organisations (and greater co-terminosity between those organisations) was also identified as a facilitator of progress.

It is notable that there was some geographic overlap between the three programmes with some pilot areas featuring in more than one programme. This might explain the similarity of some of the findings, but it might also indicate that the effort to improve integration is, in some cases at least, a long-term enterprise (although the appearance of some of the same areas in more than one programme may also be a consequence of the fact that well-organised areas have a tendency successfully to access multiple national programmes which provide financial and other forms of benefit such as prestige).

The perceived advantage of strong pre-existing relationships between stakeholders also suggests that pilots were building on inter-organisational and inter-professional relationships that preceded pilot status. Again, this points to a lengthy process and might also militate against the rapid widespread adoption of integration in those areas where this condition is not met. Even pilots with significant histories of joint working found it difficult to make headway, especially where they were attempting to implement complex changes. Some Pioneers, for example, suggested that it might take five years or longer to deliver interventions to the point that they showed demonstrable impacts.

Similar reported barriers to progress were also identified by pilots in each of the three programmes. For example, difficulties with sharing data between organisations, both in terms of system inter-operability and data governance, were a common and significant problem. This was notwithstanding the fact that information technology was seen, in theory at least, as a potential catalyst for integration. Again, there is little compelling evidence that national NHS organisations did much to address such barriers despite them featuring consistently across programmes and despite claims by national organisations that they would intervene to resolve barriers to integration.

The requirement to navigate a changing national policy context also proved challenging, with pilots in all three programmes finding that new policy initiatives cut across their objectives or added complexity to their achievement. For example, ICPs were impacted by changes to organisational arrangements for community health services and Pioneers found that national regulators were still encouraging hospitals to increase activity and their share of NHS revenue rather than work with community and social services providers to reduce such activity.

While the Vanguards were relatively closely aligned with key national objectives in their early stages (and were an important feature in the Five Year Forward View, published by NHS England in 2014 [17]), they reported that the high level of national expectations regarding their performance was burdensome, with pressure to deliver meaningful outcomes in short timescales [8]. Moreover, half-way through the programme, national policy shifted to focus on larger scale ‘Integrated Care Systems’, which limited opportunities for learning to be synthesised. However, NHS England has suggested that Vanguards’ experiences have been used to shape policy such as in relation to reducing hospital admissions [18].

Cultural obstacles were also encountered. One facet of this was the erosion of the professional identity for pilot staff as new roles were created that challenged previous long-held assumptions about skills, status and task demarcation. The Pioneers, for example, reported that differences in language and ways of working between health and social care hindered their attempts at integration [3]. Some staff in ICPs experienced a sense of professional ‘loss’ when some aspect of their role was transferred to other team members or organisations [2].

Securing and maintaining sufficient engagement of team members was also commonly a problem, particularly in relation to GPs whose activities were generally central to many integration efforts. Vanguards in particular struggled with this, with some of the so-called ‘Primary and Acute Care Systems’ having little engagement with local primary care [4,5]. This may have been partly a consequence of short deadlines for the pilot bidding processes which constrained engagement with front-line professionals. However, it was also a function of general workload pressures which for GPs had become an issue of national concern and limited their ability to engage with new ventures. Sites in all three programmes complained that insufficient resources were hampering their activities – whether a lack of funding or available workforce to free local system leaders to develop their programmes or little funding to support additional running costs while new services became established. Even the comparatively well-funded Vanguard pilots received lower levels of funding than they had expected as the pilots progressed (some received no funding in the second and third years if they were deemed not to be meeting targets for reduced emergency hospital admissions) [8]. From 2010, austerity affected NHS funding placing increased pressure on the health and care system and creating a more inhospitable environment for innovation and service change.

## What did the pilot programmes achieve?

The context of increasing financial constraint served to focus the minds of national sponsors on the impact of integrated care on hospital activity, in particular as a means by which rises in unplanned admissions to hospital might be curbed. This reduction had very commonly been identified as one of a number of objectives by all three programmes. However, it increasingly became the lens through which ‘success’ was judged by NHS leaders – something that became uncomfortable and overly reductive for pilots by the time Vanguards had been established. This focus also served to diminish the role of local authorities and voluntary sector partners who often had a broader set of success criteria.

In this regard, the integrated care programmes achieved only mixed results. ICPs resulted in significant reductions in elective admissions and outpatient appointments, which had not been an overt objective, but an increase in unplanned admissions which was the reverse of what had been intended [1]. Pioneers achieved a modestly lower rise in unplanned admissions to hospital than controls (only statistically significant in the first of two years) [7]. Vanguards also achieved modestly lower rises in unplanned admissions than controls (especially those pilots relating to care homes) but with statistically significant differences largely being seen in the last year of the programme. This concealed the fact that there was significant variation in this regard among the Vanguard areas. In some cases, their initial unplanned admission rates were higher than those of non-Vanguard areas which may have influenced their ability to reduce the rate during the pilot [10].

There is some evidence that sites that took part in both the Pioneer and Vanguard programmes were able to make more sustained reductions in the growth of unplanned admissions [19]. It is also notable that a longer-term evaluation of one Vanguard site found that a lower rate of increase in a range of measures of unplanned hospital care towards the end of a six-year period of pilot operation. The evaluators hypothesised that they may have captured the impact of sustained improvements to community care on hospital usage which may be missed in shorter periods of analysis [12].

Impacts of pilots on patient and staff experience are harder to determine across all programmes. Such data are still being collected in the Pioneers evaluation and for Vanguard pilots patient experience is part of various local evaluations of variable quality [20].

Surveys of ICP staff were generally positive about the impact of the pilots. Notably, staff suggested that communication had improved within and between organisations and that patient care had improved as a result of the pilot. Moreover, nearly half of staff directly engaged with pilots felt that their jobs had become more interesting (and only 2.4% of staff surveyed less interesting). Vanguard qualitative case studies did not explore staff perceptions of roles and outcomes directly. However, it was clear that the pilot programmes were consistent with local aspirations and a strong sense of public service ethos. Vanguards commonly were associated with developing a local sense of purpose and common vision among staff [11].

The impact of ICPs on patient experience was more mixed. Positive findings included that patients were more likely to have been told that they had a care plan and those patients in the ‘case management’ sites were more likely to report clear follow-up arrangements and to know who to contact on discharge. However, the latter group of patients also reported being less likely to see a doctor or nurse of their choice, felt less involved in care decisions and were less likely to feel that their opinions and preferences had been taken into account. The evaluators hypothesised that ICPs had the effect of ‘professionalising’ care rather than engaging with patients [1].

The negative views of ICP patients can perhaps be triangulated with the Pioneer surveys of ‘key informants’ leading pilots or involved with integration locally. Less than 10% of the latter group when surveyed in 2019 identified involving users/voluntary sector as a top priority (0% among NHS informants) and ensuring patients had a greater say in their own care was the lowest scored of ‘most important’ objectives. However, ensuring that patients/service users experienced more joined up services was consistently the top-rated objective over the previous three years of the surveys [9].

## Discussion

The last 12 years have seen a determined but restless effort to test out ways in which integrated care might best be designed and implemented in England (although this is also an international phenomenon [21]). Given this level of activity, one might question why the debate about how best to integrate health and social care in England remains unfinished business. No single programme has been able to distil key, generalisable ‘lessons’ that have then been applied subsequently. Indeed, successive programmes did little to build on one another in their conception nor to synthesise learning as they progressed.

The root cause of this absence may lie in a lack of clarity and consensus regarding the precise definition of integrated care, and the objectives of policy makers and local health and care teams. Most programmes had an ambiguity at their heart as to whether integrated care primarily related to better inter-professional working, new types of health and care organisations, or the introduction of new types of clinical interventions – or all three. It is possible that this (ambiguous) conceptualisation is misplaced and that integration is best supported by focusing, not on what is done and within what organisational construct, but on what patients and carers consider to be needed and what will best support care workers of all sorts to work effectively together. Coordination across professional and organisational boundaries will remain the key challenge and finding ways to support that may be more fruitful than designing complex integration initiatives.

In this conceptualisation, the precise ‘recipe’ for integrated care is likely to be highly context-specific and therefore generalisable lessons about ‘what integrated care should look like’ may arguably be unhelpful given that local experiences have been shaped by factors such as local leadership capacity, opportunities to make progress that are peculiar to local conditions and the shared history of stakeholders. More valuable might be the gathering of insights into the principles and processes that support staff to work together better.

In this regard, the three programmes do provide useful (although not surprising) insights. Ensuring that information systems support and do not hinder care coordination, supporting the development of local clinical and other leaders, and providing adequate resources to allow new services to be designed and implemented are all likely to aid success.

The challenge of learning from such a heterogenous group of pilots (that rightly adapted and changed over time) should not be underestimated. Similarly, more clarity about the relevant theories of change may have helped to address the ambiguity we have identified here. Looking forward, future evaluation efforts could more usefully focus upon the extent to which any particular innovation helps or hinders cross-boundary working in the service of person-centred care. Conceptualising integration as a form of *work done* by staff in collaborating may be a useful way of shifting focus from designing models to looking for simple fixes that make day-to-day work easier.

Some uncomfortable truths (at least for those wishing to make rapid progress) also need to be accepted. The pilots’ experiences suggest that integration is not a short-term project (a belief shared by many pilot participants and perhaps supported by some of the quantitative evaluation findings) [10,12]. Results have been modest and there is good reason to think that these pilots represent the best of what could be achieved in the period. The pilots were not randomly selected; they were volunteers, often passionate about integration; they often had a history of local joint working; and they received considerable support from the centre that is unlikely to be scalable across the NHS as a whole.

The period of financial austerity (and, for local authorities, significant financial cuts) also shaped the pilot programmes’ experience both in terms of the availability of resources to lubricate the service change process but also, importantly, the way in which ‘success’ was interpreted. Increasingly, pilots were seen by the NHS and its policy makers, although much less so by local government and third sector organisations, as agents for the reduction of unplanned hospital admissions (a phenomenon that was seen as increasing overall health care costs). However, it is also notable that since 2010 the share of NHS resources dedicated to primary care has declined – an outcome that is hardly supportive of a shift of care away from the hospital sector [22].

The evaluations have shown that even a modest curbing in the upward trend of unplanned admissions is not guaranteed, takes a long time, may not always be sustained, and may arguably not prove to be value for money. Erens et al. have referred to the ‘integration paradox’ – the observation that the constrained funding that clearly inhibited attempts to deliver integrated care was precisely the stimulus that drove policy makers to adopt the policies in the first place [3].

It might also be argued that the design and implementation of at least some integrated care initiatives have tended to be dominated by professional views of effective care, not always focusing on engaging with patient/user-defined needs. Efforts were made to inculcate a user focus – for Pioneers through focusing on realising a set of user-focused statements created by national patient bodies and for Vanguards through public events. However, engagement of citizens and patients receiving services in the design of the interventions was less evident. Given the importance of user engagement as a pre-requisite of effective management of people with long term diseases (a key cohort targeted by pilots) this would appear to be an important omission [23].

Looking forward, the macro context for care integration is changing. The current focus within the NHS and care system is on systemic integration through the vehicle of Integrated Care Systems. These systems cover large populations and establish voluntary governance arrangements embracing local statutory and voluntary sector agencies (although their structure, role and governance may soon be shaped by new legislation). Beneath these ‘systems’ additional partnership arrangements are being created to focus on smaller populations, known as ‘places’ and ‘neighbourhoods’, the latter involving networks of GP practices and other community and social care providers [24]. Together these changes look set to replace the last vestiges of the NHS competitive ‘market’ with a system based more on collaboration, joint planning and shared resources within geographic areas. Proposals to formalise these arrangements through legislation have recently been published by the Government in the form of a White Paper [13].

It is possible that this contextual change may create a more supportive environment for local health and care teams seeking to work in an integrated way by mitigating some organisational conflicts that have hitherto acted as obstacles faced by pilots while operating in a ‘disintegrated’ system. Furthermore, shifting focus from ‘integrated care’ to ‘work required to integrate’ might provide a vehicle through which nascent partnerships can diagnose their problems and begin to design effective solutions.

The Covid-19 pandemic has also acted as a significant catalyst for service change, some of which might also support better integration assuming it becomes a permanent feature of the health and care system. Under emergency conditions (and, in the UK, with a relatively strong nationally coordinating and directing function) a sense of ‘system’ has been accentuated with clearer integration between health and care organisations and better data sharing. This has also driven forward the deployment of tools and approaches that may support wider efforts at integration, such as an increase in the availability and use of technology to support primary care and specialist communications and the clearer designation of hospital discharge coordination roles are likely to prove helpful to inter-organisational working [25].
